# Anion-Specific Water Interactions with Nanochitin: Donnan and Osmotic Pressure Effects as Revealed by Quartz Microgravimetry

**DOI:** 10.1021/acs.langmuir.1c01585

**Published:** 2021-09-14

**Authors:** Soo-Ah Jin, Saad A. Khan, Richard J. Spontak, Orlando J. Rojas

**Affiliations:** † Department of Chemical & Biomolecular Engineering, North Carolina State University, Raleigh, North Carolina 27695, United States; ‡ Department of Materials Science & Engineering, North Carolina State University, Raleigh, North Carolina 27695, United States; § Bioproducts Institute, Departments of Chemical & Biological Engineering, Chemistry and Wood Science University of British Columbia, Vancouver V6T 1Z3, Canada; ∥ Department of Bioproducts and Biosystems, Aalto University, Espoo 02150, Finland

## Abstract

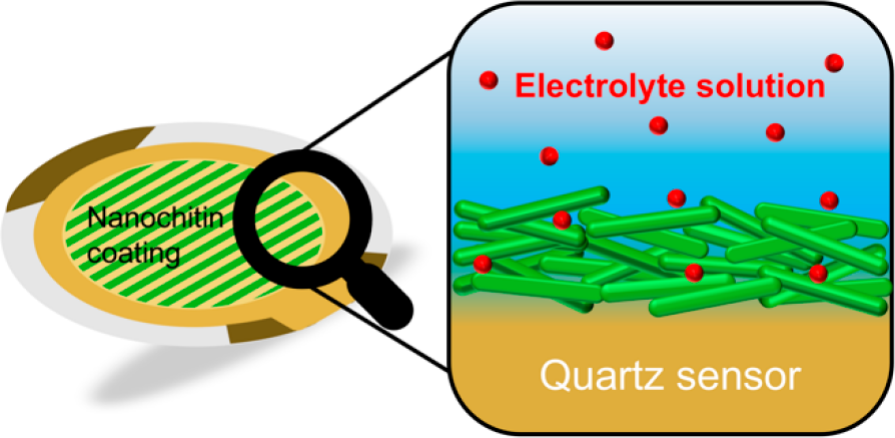

The development of new materials emphasizes greater use of sustainable and eco-friendly resources, including those that take advantage of the unique properties of nanopolysaccharides. Advances in this area, however, necessarily require a thorough understanding of interactions with water. Our contribution to this important topic pertains to the swelling behavior of partially deacetylated nanochitin (NCh), which has been studied here by quartz crystal microgravimetry. Ultrathin films of NCh supported on gold-coated resonators have been equilibrated in aqueous electrolyte solutions (containing NaF, NaCl, NaBr, NaNO_3_, Na_2_SO_4_, Na_2_SO_3_, or Na_3_PO_4_) at different ionic strengths. As anticipated, NCh displays contrasting swelling/deswelling responses, depending on the ionic affinities and valences of the counterions. The extent of water uptake induced by halide anions, for instance, follows a modified Hofmeister series with F^–^ producing the highest swelling. In marked contrast, Cl^–^ induces film dehydration. We conclude that larger anions promote deswelling such that water losses increase with increasing anion valence. Results such as the ones reported here are critical to ongoing efforts designed to dry chitin nanomaterials and develop bio-based and sustainable materials, including particles, films, coatings, and other nanostructured assemblies, for various devices and applications.

## Introduction

The field of bio-derived materials is rapidly developing due to their important attributes, such as sustainability, biodegradability, and eco-friendliness. Numerous biopolymer archetypes can be chemically and/or mechanically processed to yield (nano)structures that can greatly broaden their utility in advanced materials. With increasing environmental awareness accompanied by stringent regulations, many industries have begun incorporating bio-based materials into products. Nanopolysaccharides, including those derived from cellulose and chitin, are of particular importance because of their versatility and widespread use in rheological control,^1−4^ mechanical reinforcement,^5−8^ and many other technological applications.^9−11^ Highly crystalline nanochitin (NCh), derived from bioreosurces that include marine shells, insects, and fungi, is gaining considerable attention due to its attributes, some of which compare favorably with those of nanocelluloses. Some prominent uses include Pickering emulsion stabilization,^12−15^ drug delivery,^16−19^ and food packaging.^20−22^ While NCh can be readily dispersed in an aqueous environment due to its inherent surface charge, its association and stability are susceptible to changes in the medium. For instance, variations in pH or ionic strength are known^13,23^ to affect the colloidal stability of NCh suspensions. Therefore, a study of related interactions is critical to not only achieving a better fundamental understanding of NCh behavior in different environments but also efficiently designing NCh-based materials, including composites.

With regard to surface interactions, previous reports^24−29^ of cellulose nanofiber (CNF) under given suspension conditions are particularly noteworthy. For instance, Ahola et al.^24^ have explored the effects of charge density, ionic strength, and pH on the swelling and surface interactions of CNF thin films in aqueous media. As can be expected from colloidal chemistry and observations of film deswelling and stiffening, highly charged (HC) CNF is found to respond more sensitively to changes in ionic strength compared to low charge (LC) nanofibrils. While both classes of CNF films undergo reversible swelling, HC CNF sorbs more water than LC CNF at high pH. These effects are explained by the fact that the carboxyl groups deprotonate and dissociate, introducing negative surface charges that promote interactions with water and thus increase the swelling capacity. Depending on the colloids present in an aqueous system, ions can exhibit vastly different interactions with solutes and neighboring water molecules, thereby affecting the overall system stability.^30^ Inorganic salts are able to alter the stability of macromolecules, including proteins, in aqueous media,^31−34^ and recent studies have established that electrolytes alter the lyotropism of cellulose nanocrystal (CNC) in aqueous suspensions,^35,36^ as well as in the subsequent structuring of dried CNC films.^37^ Despite the fact that electrolytes are of tremendous physiological and biological importance, their effect on NCh interactions in aqueous media still remains largely unknown. Interactions of NCh with macromolecular proteins, such as bovine serum albumin^8,19,38^ and chitinase,^8^ have, however, been studied by quartz crystal microgravimetry with dissipation (QCM-D).

More than a century ago, Hofmeister^39^ described protein stability in aqueous media according to the ions present, which were categorized on the basis of their effectiveness to precipitate proteins. This classification is popularly referred to as the Hofmeister series. Protein precipitation is known to depend on the hydration ability of ions, since they exert an indirect influence by changing the extent of interaction with water molecules. Ions with high hydration capabilities are designated as “kosmotropes” (water-structure makers), while those with low hydration capabilities are termed “chaotropes” (water-structure breakers). Kosmotropic ions are responsible for ordering water molecules in, *e.g.*, clusters, thereby reducing protein solubility and promoting crystallization. In contrast, chaotropic ions prevent water cluster formation and induce protein denaturation and increase protein solubility. Such phenomena associated with protein stability and solubility introduce “salting-out” and “salting-in” effects, respectively. An ionic series, or its slightly modified version, has been widely observed in nonbiological systems, giving rise to the “law of matching water affinity.”^31−33^ For example, Phan-Xuan et al.^40^ have confirmed that (i) the aggregation behavior of CNC is sensitive to the type and valence of cations present in the suspension and (ii) the critical aggregation concentration induced by cations follows the Hofmeister trend. Indeed, over the past quarter century, the Hofmeister series has been successfully applied^41−48^ to a broad spectrum of synthetic and bio-based macromolecules to describe their interactions with ions in aqueous media.

In this study, we investigate the anion-specific swelling/deswelling behavior of NCh thin films in the presence of different electrolytes at several concentrations. After immersion in water, NCh forms uniform and stable layers on gold resonators, used as supports in QCM-D experiments. This analytical technique is well-suited for elucidating surface interactions given its sensitivity to changes in water coupling, as measured by changes in resonance frequency and energy dissipation. The films are remarkably stable (under long time and cyclic electrolyte solution conditions) and display distinctive swelling/deswelling behavior, depending on the counterion type and concentration. This study fills a missing and important knowledge gap that currently exists in our understanding of NCh and its widespread use in relation to processes that require aqueous media, as is most often the case.

## Experimental Section

A stock NCh aqueous suspension was prepared following our earlier protocol.^23^ Briefly, α-chitin was extracted from fresh crabs (*Callinectes sapidus*) purchased from a local harbor market. Residual crab shells were subjected to purification, and the chitin flakes obtained were subsequently treated with 33 wt % NaOH(aq) solution at 90 °C for 4 h (25 mL/g liquid-to-solid relation). This process yielded a partially deacetylated chitin suspended in water, which was thoroughly washed with deionized (DI) water to achieve neutral pH. In contrast to acid-soluble chitosan that possesses a typical degree of deacetylation of at least 55%, the degree of deacetylation of the chitin employed here (which did not dissolve in acid) was ≈27.3%. For mechanical nanofibrillation, the deacetylated chitin was dispersed in DI water at a concentration of 0.2 wt %, and the pH of the suspension was lowered to 3 through the introduction of acetic acid. The suspension was then homogenized in a high-speed blender (T-25 Ultra-Turrax Digital Homogenizer, IKA, Germany) at 10 000 rpm at ambient temperature for 5 min, followed by pulsed ultrasonication (Sonifer 450, Branson Ultrasonics Co., Danbury, CT) at 50% amplitude with cycles of 5 s on and 2 s off for 40 min. The resultant suspension was then centrifuged at 10 000 rpm for 5 min to remove large particles, and the supernatant was collected as the final NCh suspension. Several electrolyte solutions—1.0 M NaF (Sigma-Aldrich), 1.0 M NaCl (VWR Chemicals), 1.0 M NaBr (Sigma-Aldrich), 1.0 M NaNO_3_ (VWR Chemicals), 0.5 M Na_2_SO_4_ (Merck Millipore Chemicals), 0.5 M Na_2_SO_3_ (Merck Millipore Chemicals), and 0.5 M Na_3_PO_4_ (Sigma-Aldrich)—were prepared and diluted to desired concentrations. All the electrolyte suspensions were filtered (0.45 μm pore size) to remove large particles and degassed by using a bath sonicator to remove air bubbles prior to testing. The pH of all the electrolyte solutions was ∼5, similar to that of DI water.

The NCh stock suspension was diluted to 0.01 wt % and vigorously stirred prior to spin coating onto gold-coated quartz sensors (Q-Sense, Gothenburg, Sweden). Before this process, the gold AT-cut quartz crystal sensors (measuring 14 mm in diameter) were cleaned by immersion in 20 wt % NaOH(aq) solution for 30 s, followed by rinsing with DI water, drying with nitrogen gas, and exposure to UV/ozone for 20 min. A droplet (100 μL) of NCh suspension was deposited onto the crystal sensor and spin-coated (WS-650SX-6NPP/LITE, Laurell Technologies Co., North Wales, PA) at 3000 rpm for 60 s to form a thin layer. The coated crystal sensor was finally heat-treated at 80 °C for 10 min and subsequently immersed in DI water to achieve equilibration overnight before analysis. The morphology of NCh films spin-coated onto gold sensors was confirmed by AFM (Asylum MFP-3D) operated in AC mode at ambient conditions. Multiple areas were imaged to ensure full coverage prior to QCM-D measurements (Q-Sense E4, Gothenburg, Sweden). Changes in the adsorbed mass and viscoelastic properties of the film layer were measured simultaneously by monitoring the change in frequency (Δ*f*) and the energy dissipation (*D*), respectively, at the fundamental resonance frequency (5 MHz) and its overtones (at 15, 25, 35, 45, 55, and 75 MHz). The interpretation of the data followed protocols described in detail elsewhere.^49−51^ For each new measurement, DI water was passed through the chamber for at least 2 h to ensure a stable signal (equilibrium condition). The flow rate was set to 0.1 mL/min, and each measurement was repeated in triplicate (average values are reported). The extent of deswelling (effective mass loss) was related to the shift in the resonance frequency according to the Sauerbrey equation for rigid films:^51^
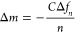
1where the mass change per unit surface area (Δ*m*) is assumed to be linearly proportional to the frequency shift (Δ*f*_*n*_). The parameters (*C* and *n*) represent the mass sensitivity (17.7 ng Hz^–1^ cm^–2^ for a 5 MHz crystal) and resonance overtone number (*n* = 1, 3, 5, 7, 9, and 11), respectively. In this work, we only considered the third overtone (*n* = 3) and refer to Δ*f*_3_ as simply Δ*f*. The value of *D*, defined as the inverse of the quality (*Q*) factor, is related to the inverse of the oscillatory decay time.

## Results and Discussion

**Figure 1:**
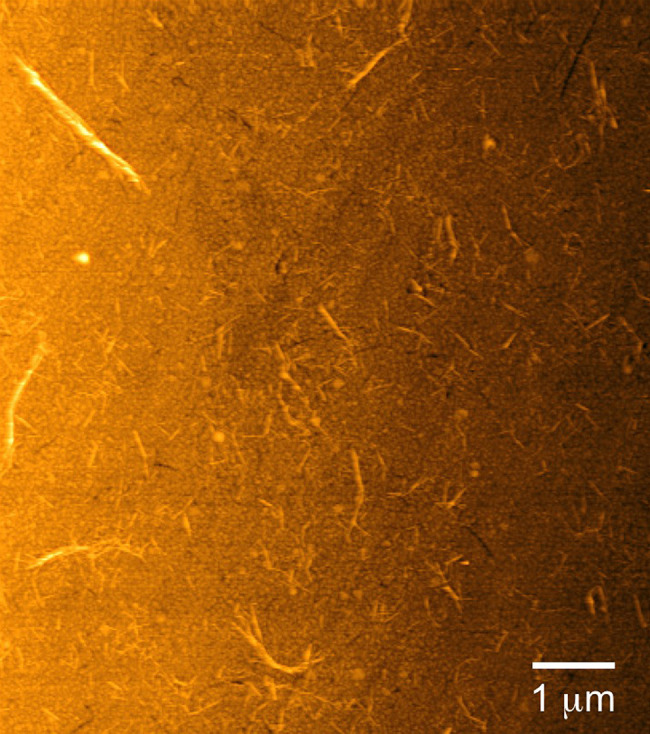
AFM height image of NCh deposited from a 0.01 wt % aqueous suspension onto the surface of a gold sensor for QCM-D measurements.

**Figure 2:**
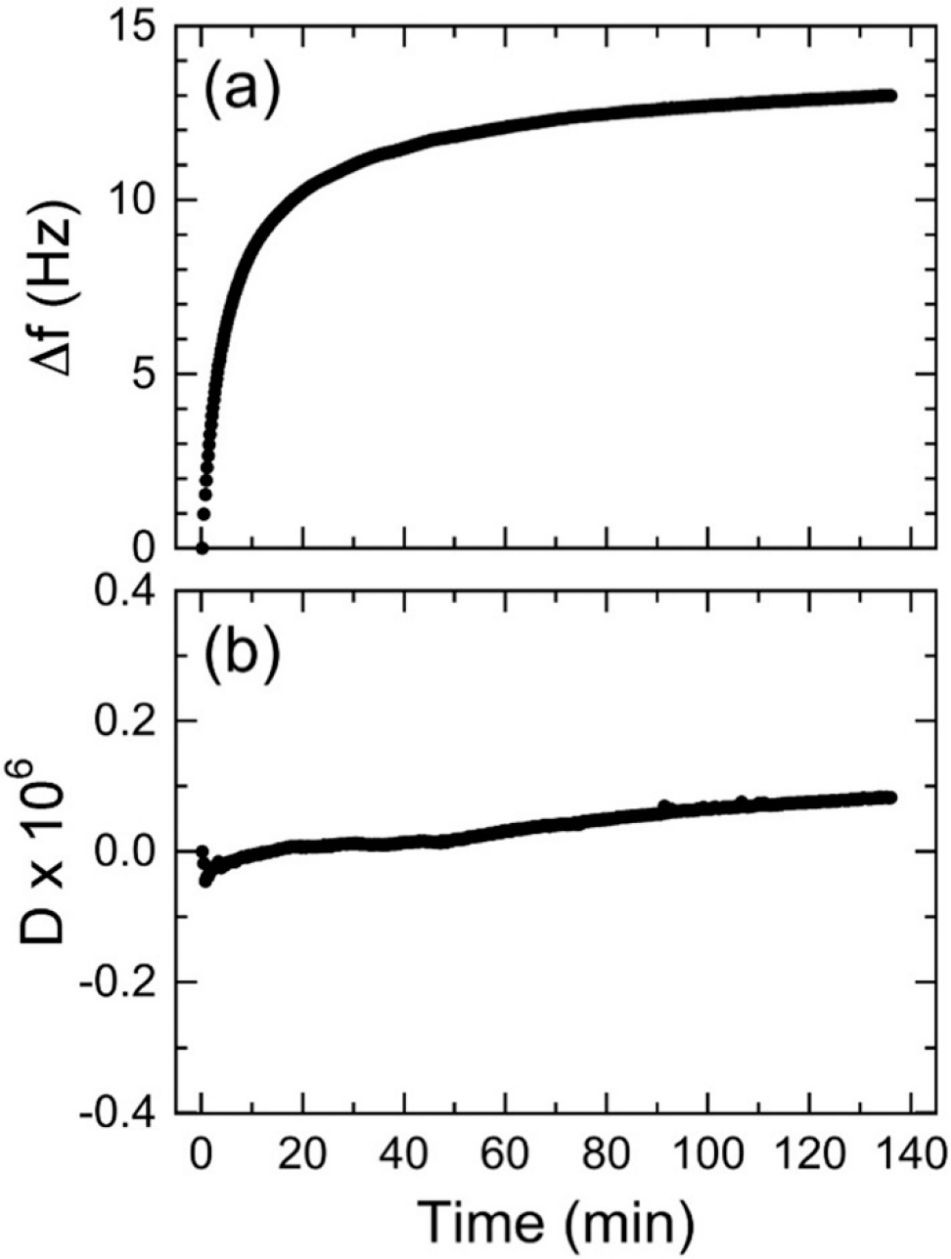
Representative QCM-D data (at the *n* = 3 overtone) displaying the (a) frequency shift (Δ*f*) and (b) energy dissipation (*D*) of a NCh thin film adsorbed to the surface of a gold sensor and subsequently exposed to DI water, which is injected into the chamber at 0 min.

In agreement with the results reported by Bai et al.,^12^ the aspect ratio of the NCh nanofibers in aqueous suspension is measured here to be ∼15 (166 ± 25 nm in length and 11 ± 2 nm in width, according to AFM measurements). According to the AFM height image displayed in Figure 1, the NCh nanofiber film appears to be evenly distributed over the entire gold sensor, uniformly covering the surface. Loose nanofibers randomly oriented on, but not necessarily adhered to, the NCh thin film are also evident. In addition to verifying the extent of NCh surface coverage by AFM, we have performed QCM-D tests in DI water to discern the real-time (*t*) stability, as well as the swelling capacity of the NCh thin film. The initial response of the NCh film to DI water is presented in Figure 2, in which DI water is introduced into the chamber that initially contained the NCh film. Soon after time *t* = 0 min, Δ*f* in Figure 2a increases sharply during the first ∼20 min before starting to display evidence of a signal plateau. Fitting an empirical saturation curve of the form Δ*f* = *At*/(*B* + *t*) to the data reveals a plateau or limiting value at 13.39 ± 0.01 Hz. While an increase in Δ*f* is typically associated with mass loss due to, for instance, desorption of water or removal of loosely bound nanofibers (such as those visible in Figure 1), other system characteristics must be considered before interpreting frequency responses such as these (discussed later). The associated energy dissipation (*D*) curve pictured in Figure 2b remains stable near zero throughout the experiment, which implies that the NCh film is tightly and rigidly attached to the sensor surface. Cyclic exposure to water, accompanied by complementary AFM imaging, confirms the retention of full surface coverage even after the NCh film is kept in contact with water for prolonged exposure times. From these observations, we conclude that the the films are suitable for further analyses.

### Halide Anions and Their Affinity with NCh

**Figure 3:**
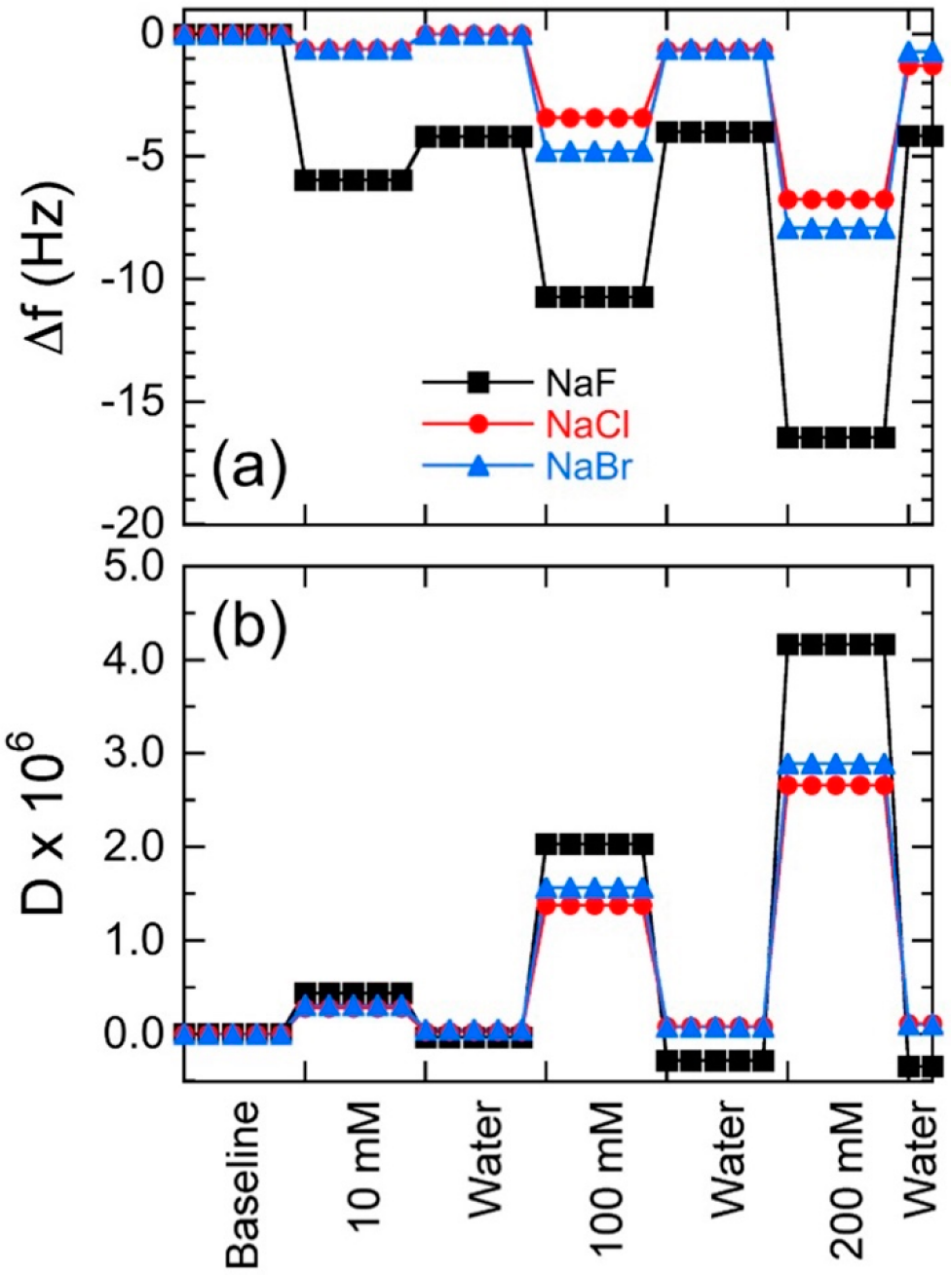
Normalized QCM-D data (at the *n* = 3 overtone) indicating apparent changes in (a) Δ*f* and (b) *D* for NCh thin films upon exposure to three different halide electrolytes (with a Na^+^ cation, see legend) at three different concentrations. The chamber is rinsed with DI water (pH 5) after each electrolyte exposure at 23 °C.

We first consider the effect of halide anions, namely, F^–^, Cl^–^, and Br^–^, at different concentrations on the swelling behavior of NCh thin films. Real-time QCM-D responses corresponding to different Na-paired halide solutions are provided in Figure S1. In these studies, the electrolyte concentration is increased discretely and sequentially from 0 to 200 mM. After each electrolyte exposure the chamber is rinsed with background medium (DI water), while Δ*f* and *D* are continuously monitored, in the absence of electrolyte. The average values of Δ*f* and *D*, collected after signal stabilization upon introduction of each electrolyte at each concentration, are reported in parts a and b, respectively, of Figure 3. The QCM-D profiles are normalized so that the baseline initially zeroes Δ*f* and *D*. Two signature features are immediately evident from Figure 3. As the electrolyte concentration is increased, the vibration frequency shifts to smaller values (yielding a negative frequency shift, Δ*f* < 0 in Figure 3a). On the other hand, the dissipation increases (yielding a positive dissipation shift, *D* > 0 in Figure 3b). These changes are more pronounced for halide anions in the order F^–^ > Br^–^ > Cl^–^. In addition, the degree of hysteresis, or irreversibility, in Δ*f* (as assessed by the difference in Δ*f* relative to its initial baseline at 0 Hz upon sequential reintroduction of electrolyte-free DI water) consistently appears to be most pronounced for solutions containing F^–^. On the basis of the Sauerbrey relationship in eq 1, a reduction in Δ*f* generally indicates an increase in the mass of the thin film. We posit that two molecular-level mechanisms can explain this observation: (i) electrostatic interactions between positively charged NCh and mobile anions in the solutions, which may also affect electrostatic interactions between chitin nanofibers, and (ii) concurrent film dimensional changes caused by accompanying changes in osmotic pressure and water sorption. Electrolyte-driven water swelling is further anticipated to yield NCh thin films that are not as rigid as the ones originally deposited on the resonator. This expectation, along with the postulated mechanisms mentioned above, are corroborated in Figure 3b by the increasing *D* profiles, which imply that the NCh films soften and become more viscoelastic when exposed to electrolytes in the aqueous media.

**Figure 4:**
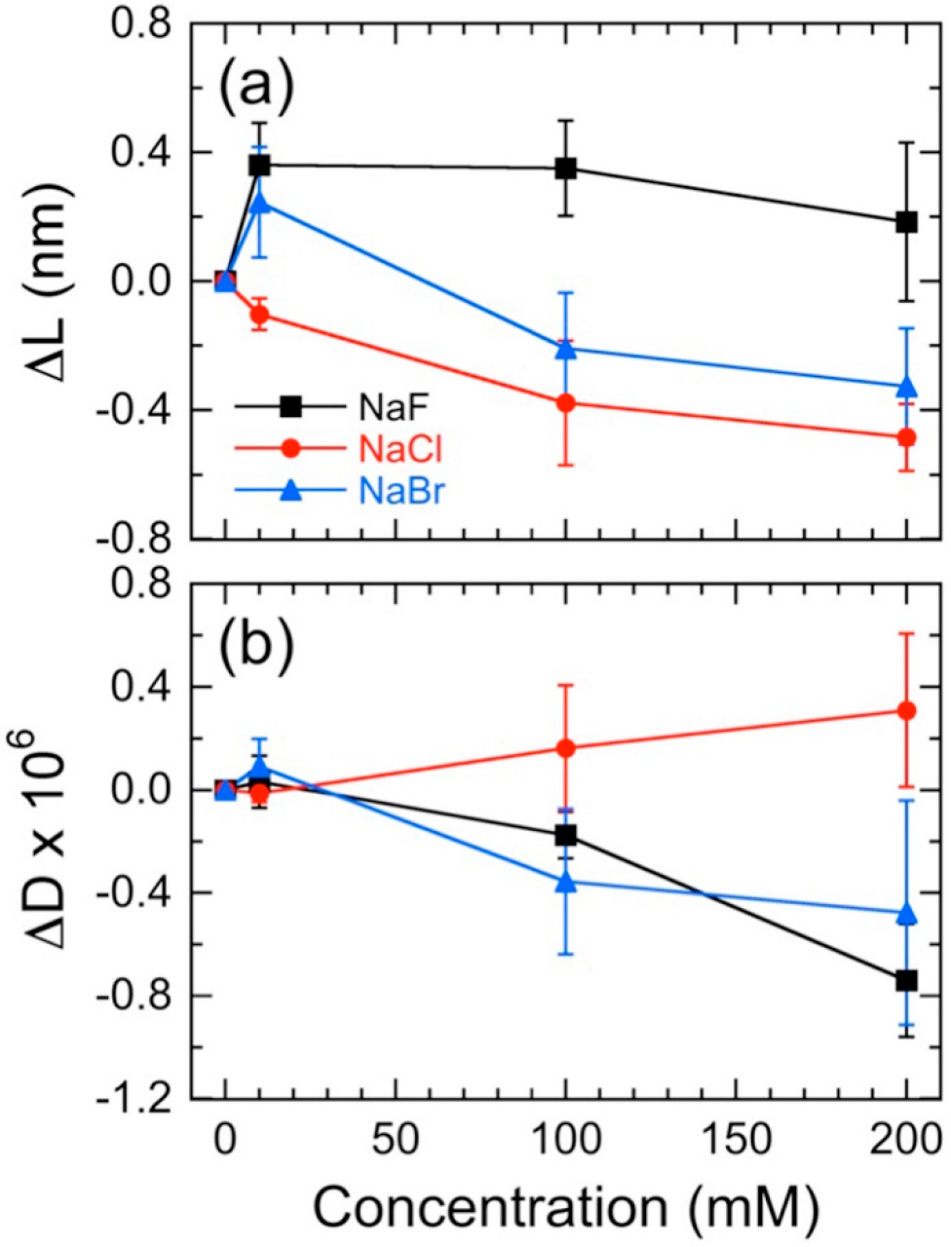
Effects of halide anions on (a) apparent thickness change (Δ*L*) and (b) corrected viscoelastic properties (Δ*D*) of NCh thin films as functions of electrolyte concentration and salt type (see legend). Bulk effects are eliminated by subtracting analogous results from the uncoated sensor. Values of Δ*L* are extracted from Δ*f*_corr_ in conjunction with eq 1. Error bars represent standard errors in the data, and solid lines serve to connect the data.

However, an important factor that cannot be overlooked in this physical interpretation is the bulk effect generated by a change in the environment.^24,29^ The QCM is sensitive to physical and chemical surface changes at the nanoscale. An apparent Δ*f* can be caused by the response of a film to the surrounding medium or, alternatively, by a change in the medium itself (for instance, its viscosity). It is therefore crucial to verify the origin of Δ*f* with certainty. To discern the magnitude of bulk (*e.g.*, viscosity) effects, we performed the same analysis as above but with just the pristine (uncoated) sensor (*cf.*Figure S2). The resulting frequency shift (Δ*f*_surr_) measured at each electrolyte concentration was then subtracted from the value of Δ*f* determined for the NCh film. The net difference (Δ*f*_corr_ = Δ*f* – Δ*f*_surr_) identifies the frequency shift associated with changes in the film. In similar fashion, *D*_surr_ is likewise measured to ascertain the magnitude of the contribution arising from changes in the aqueous medium, and the resultant value of Δ*D* (=*D* – *D*_surr_) in conjunction with Δ*f*_corr_ satisfies the Sauerbrey empirical condition^49^ ( < 4 × 10^–7^ Hz^–1^) over the electrolyte concentration range examined. This outcome confirms that the deposited NCh thin films investigated here remain sufficiently rigidly attached to the sensor throughout each sequential test. Moreover, since the Sauerbrey condition remains valid throughout our study, we can analyze Δ*m* from Δ*f*_corr_ according to eq 1, with the assumption that the mass density of dry NCh (reported^52,53^ as 1.425 g/cm^3^) remains constant, to evaluate the apparent change in film thickness (Δ*L*) induced by exposure to different types and concentrations of electrolyte solutions. Calculated values of Δ*L* and Δ*D* are presented as functions of electrolyte concentration and type in Figure 4, parts a and b, respectively.

While the raw QCM-D data presented in Figure 3a implied that the NCh film consistently swelled in the presence of all the electrolyte solutions under consideration, correcting for the salt-induced change in solution viscosity reveals that only films exposed to solutions containing F^–^ swell consistently (Δ*L* > 0) over the entire concentration range examined (Figure 4a). In this case, films initially increase in average thickness to ∼0.4 nm at 10 mM NaF but then decrease to ∼0.2 nm at 200 mM NaF. Within experimental uncertainty, however, these measured values do not change substantially. While addition of 10 mM NaBr similarly, but not as profoundly, increases the film thickness (to ∼0.2 nm), the film deswells and Δ*L* becomes negative with increasing electrolyte level. As in the case of NaF, the experimental uncertainty in the data suggests that there is little difference between measurements collected at 100 and 200 mM NaBr. In marked contrast to the response of the NCh films to NaF and NaBr electrolyte solutions, the change in film thickness at 10 mM NaCl is initially negative and monotonically decreases further with increasing Cl^–^ concentration, until it reaches about −0.5 nm at 200 mM NaCl. From these measurements, we surmise that the presence of F^–^ and Br^–^ enhances the interactions between NCh thin films and their environment (but to different extents), thereby inducing film swelling. Interactions with F^–^ appear to be significantly greater and persist from low to high salt concentration, whereas such interactions with Br^–^ are limited to only low NaBr concentration levels. At high concentrations of Br^–^ and at all concentrations of Cl^–^, the films compress to reduce exposure of NCh to these two electrolytes.

Two types of competing interactions are expected to exist in this system: (i) electrostatic interactions between halide anions and NCh and (ii) hydrogen-bonding interactions between water and NCh. Recall that, during synthesis, acetamide groups of NCh are partially deacetylated to yield amine groups, which are responsible for electrostatic interactions with their halide counterions.^12,13,23^ Since the pH of the electrolyte solutions was ∼5 and the p*K*_a_ of amine in water is 6.3, the majority of the amines are in their protonated state. As a result, the NCh thin films remain positively charged throughout their exposure to the electrolyte solutions, and their surfaces could form complexes with halide anions. The interaction between halide anions and charged surfaces (hydrophobic and hydrophilic) has been previously investigated.^46,54,55^ Tuladhar et al.^54^ have indirectly studied the interaction between halide anions and positively charged alumina by monitoring the intensity of hydrogen bonding between interfacial water molecules and adjacent aluminol groups. From their spectroscopic results, they conclude that anion affinity at the alumina surface follows the order F^–^ ≫ Br^–^ > Cl^–^ > I^–^, which is a slightly altered version of the conventional Hofmeister series (F^–^ > Cl^–^ > Br^–^ > I^–^). The smaller and more electronegative ions (F^–^) possess stronger affinity to alumina and are thus more effective at screening positive surface charges than the larger and less electronegative ones (I^–^), in favorable agreement with other independent studies^46,56,57^ conducted with hydrophilic surfaces. As a result, F^–^ disrupts the arrangement of water molecules along the surface, whereas Br^–^, Cl^–^, and I^–^ anions displace interfacial water molecules to a lesser extent.

These previously reported results appear to be analogous to the present system, since the change in NCh film thickness follows the same modified Hofmeister trend. The favorable affinity of halide anions with respect to NCh is immediately evident from the real-time QCM-D response (*cf.*Figure S1a). As mentioned earlier, Δ*f* fails to return to its original reference value of 0 Hz in the presence of F^–^ anions even after the chamber is repeatedly rinsed with NaF solution and DI water. This observation suggests that at least some of the F^–^ ions form strong ionic interactions with the NCh film and remain attached to the film surface after multiple rinse cycles. On the basis of the Hofmeister ranking, F^–^ is a strongly hydrating anion (kosmotrope) surrounded by water molecules. We therefore speculate that F^–^ is naturally drawn to the hydrophilic NCh film surface where it accumulates on the basis of the “like seeks like” rule. In addition to direct adsorption, F^–^ ions are capable of altering the arrangement of, and consequently freeing, interfacial water molecules. These liberated water molecules can subsequently diffuse into and interact with NCh via hydrogen bonding, since NCh possesses abundant hydroxyl groups. These synergistic interactions between F^–^ and NCh, in addition to interactions between water and NCh, result in swelling, as evidenced by the positive change in film thickness in Figure 4a. In contrast, Δ*f* in Figure S1a returns to its reference state after cycling with larger and less electronegative anions (Br^–^ and Cl^–^) and DI water. Interestingly, despite their opposite charge, Cl^–^ and Br^–^ are less attracted to the protonated amines of NCh, in which case they do not necessarily serve as counterions due to their facile removal upon rinsing. Although Br^–^ ions initially promote mass uptake and film swelling at 10 mM, an increase in salt concentration yields mass loss and film compression, while Cl^–^ possesses the lowest affinity for the NCh film but is responsible for the greatest (negative) ion-induced change in film thickness.^58^

Since NCh is in equilibrium with water before the electrolytes are added to the QCM chamber, mass loss and concomitant thickness reduction can be explained by the diffusion of water molecules from the film to the surroundings to promote deswelling due to water expulsion. If the NCh film is treated as a semipermeable membrane, molecules or ions can permeate through the layer depending on the driving force. Due to the presence of protonated amines in the NCh film at the pH level maintained here, NCh possesses permanently bound positive charged groups. These charges create an imbalance in the ionic distribution on either side of the NCh film, *e.g.*, the Donnan effect.^59^ In our tests, we further intensify this unequal ionic charge distribution by introducing electrolytes that can neutralize the amines along the water/film interface, thereby resulting in an increase in osmotic pressure that reduces water uptake and triggers water molecules to leave the NCh film to compensate for the ion imbalance. The Donnan effect can be helpful to explain why the NCh thin film swells in the presence of F^–^ ions. As alluded to earlier, F^–^ possesses a strong affinity for and directly adsorbs on the positively charged NCh surface. Due to such surface complexation, more negatively charged species reside on the water side of the NCh film and promote an unequal distribution of anions across the film thickness. As a consequence, water molecules permeate into the film to mitigate the imbalance and, in the process, swell the film. Despite having the same monovalent negative charge, each halide ion uniquely interacts with NCh due to different degrees of ionic affinity. So far, the order of ionic affinity that we have observed to describe interactions with NCh follows a modified Hofmeister series (F^–^ > Br^–^ > Cl^–^), and the precise nature of these ionic interactions causes both swelling and deswelling of NCh thin films.

### Affinity of Multivalent Anions with NCh

**Figure 5:**
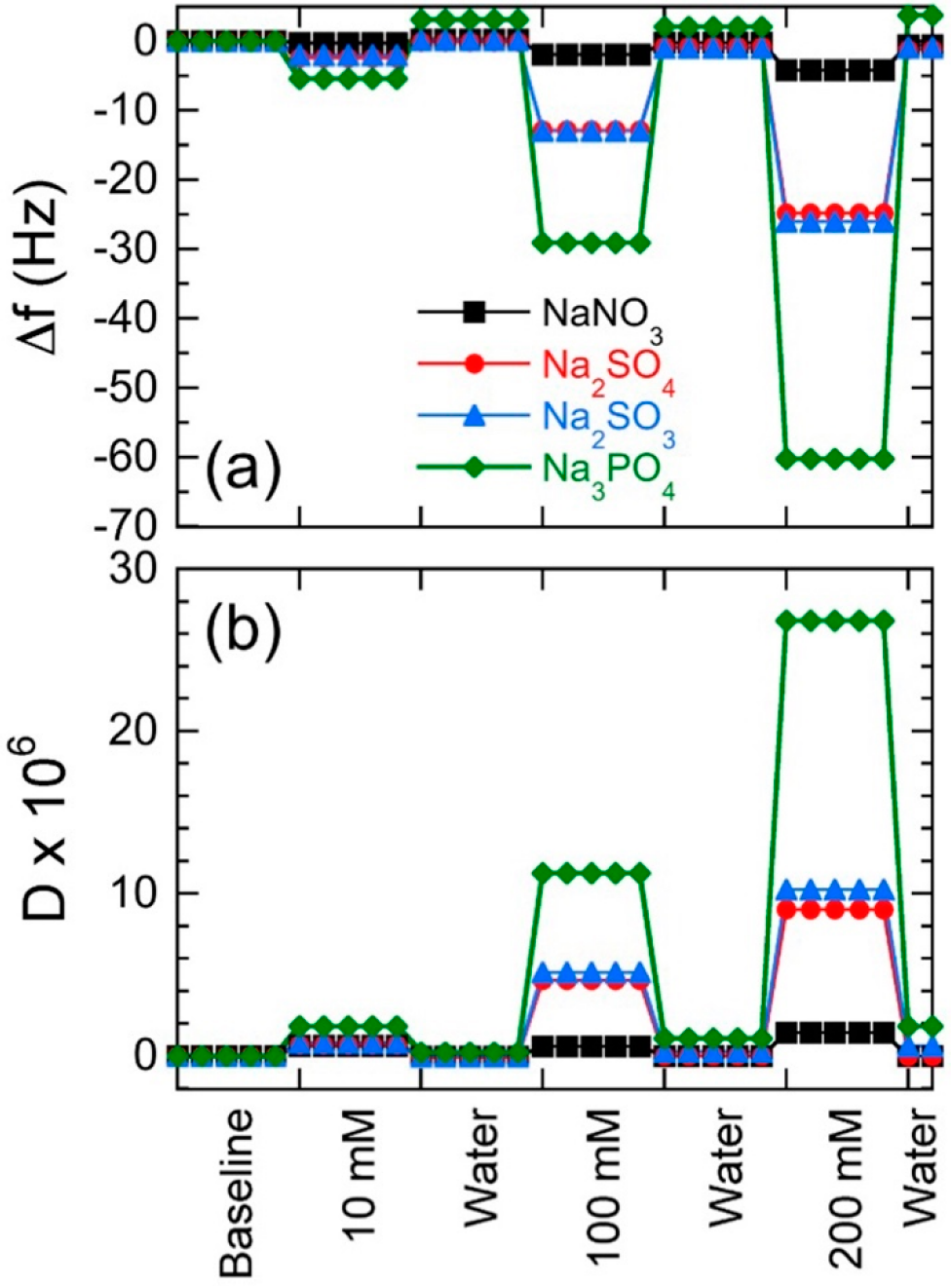
Normalized QCM-D data (at the *n* = 3 overtone) indicating the apparent changes in (a) Δ*f* and (b) *D* for NCh thin films upon exposure to four different multivalent electrolytes (with Na^+^ cations, see legend) at three different concentrations. The chamber is rinsed with DI water after each electrolyte exposure.

**Figure 6:**
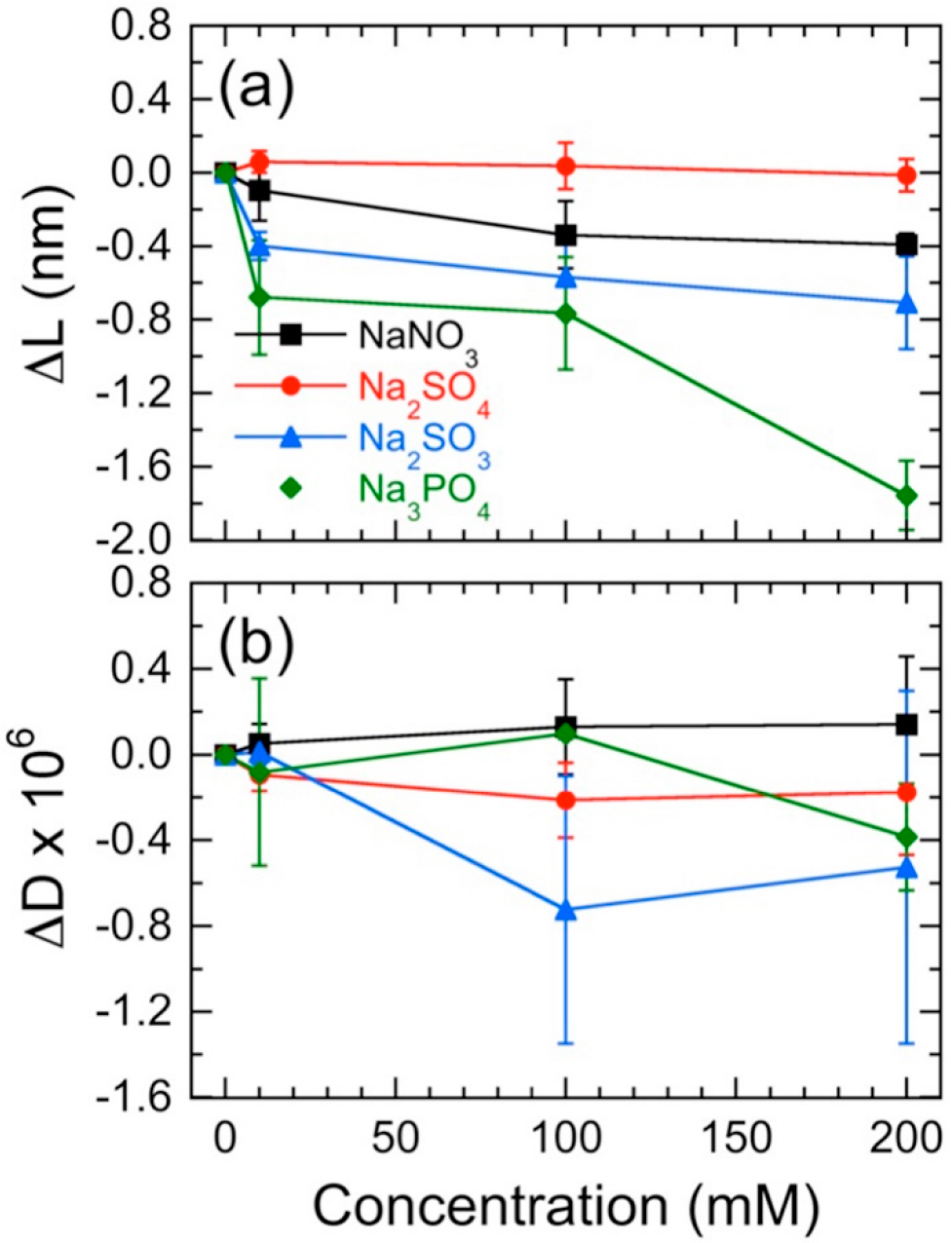
Effects of multivalent anions on (a) Δ*L* and (b) Δ*D* of NCh thin films as functions of electrolyte concentration and salt type (see legend). Bulk effects are eliminated by subtracting analogous results from the uncoated sensor. Values of Δ*L* are extracted from Δ*f*_corr_ in conjunction with eq 1. Error bars represent standard errors in the data, and solid lines serve to connect the data.

In addition to elucidating the interaction between halide anions and NCh, we extend our QCM-D tests to ascertain how NCh interacts with bulkier and multivalent anions. For this purpose, we examined electrolyte solutions containing four different anions (NO_3_^–^, SO_4_^2–^, SO_3_^2–^, and PO_4_^3–^), each paired with the balanced number of Na cations. As-measured QCM-D sensograms of NCh films (for Δ*f* and Δ*D*) are provided in Figure S3a,b, and the normalized analogs are included in Figure 5. Frequency shifts are apparent to different extents in Figure 5a upon introduction of the electrolyte solutions, with the reductions in Δ*f* becoming increasingly more pronounced as the anion valence and concentration increase. Variations in Δ*f* and *D* associated with electrolyte-induced changes in the bulk medium (viscosity) are again discerned from measurements performed with uncoated sensors (see Figure S4a,b). First, we note that the corresponding values of  continue to satisfy the Sauerbrey relationship so that values of Δ*L* can be extracted from Δ*f*_corr_ according to eq 1, and we can safely presume that the NCh remains rigidly attached to the surface in the presence of all the electrolyte solutions. Calculated values of Δ*L* and Δ*D* are displayed for comparison in Figure 6, parts a and b, respectively. Two features in Figure 6a immediately distinguish these results collected for multivalent anions from those obtained for monovalent anions: (i) none of the multivalent anions promotes NCh film swelling and (ii) the PO_4_^3–^ ion consistently produces the greatest extent of film deswelling (reaching Δ*L* ≈ −1.8 nm at 200 mM Na_3_PO_4_). On the basis of our previous discussion with regard to monovalent anions, we conclude that these multivalent anions do not interact to any significant extent with the NCh thin films, in which case the Donnan effect regulates water migration from the films, leading to film deswelling. Another interesting finding is that the dependence of Δ*L* on electrolyte concentration for monovalent NO_3_^–^ is almost identical to that of monovalent Cl^–^, which provides a bridge between the two electrolyte families investigated here.

As for the other three multivalent anions, Δ*L* monotonically decreases with increasing concentration except for the case of SO_4_^2–^. Despite exhibiting kosmotropic ion behavior, SO_4_^2–^ has negligible influence on NCh films, even at the highest concentration tested. According to our hypothesis for halide anions, SO_4_^2–^ ions should be drawn to the hydrophilic NCh surface on the basis of their anticipated hydrating efficacy, but this behavior is not observed. Unexpectedly weak hydration, which was tested additional times to ensure reproducibility, implies that another factor must be considered relative to other larger and multivalent anions, as well as monovalent halide anions, to explain how the film thickness is affected to different degrees depending on the anion. The non-oxygen species in three of the polyatomic anions possess valences of either +4 or +5, whereas the valence of S in SO_4_^2–^ is the highest (+6). Unlike sulfite (SO_3_^2–^) with one double-bonded O atom and two single-bonded oxyanions, sulfate (SO_4_^2–^) consists of two double-bonded O atoms and two single-bonded oxyanions, making it considerably bulkier than SO_3_^2–^ with a more spatially even charge distribution. Another distinguishing aspect of SO_4_^2–^ that can help explain its weak hydration is that its dimensionless friction coefficient (0.24) is smaller than those of NO_3_^–^ (0.27) and the halide anions (∼0.25). Systematic mass loss and thickness compression are evident for the remaining multivalent anions, and the results clearly follow a consistent order of PO_4_^3–^ ≫ SO_3_^2–^ > NO_3_^–^ over the entire concentration regime. On the basis of this result, we conclude that NCh film deswelling is sensitive to the valence state of the anions with trivalent anions being more efficient at displacing water from the film than monovalent and divalent anions at the same electrolyte concentration.

**Figure 7:**
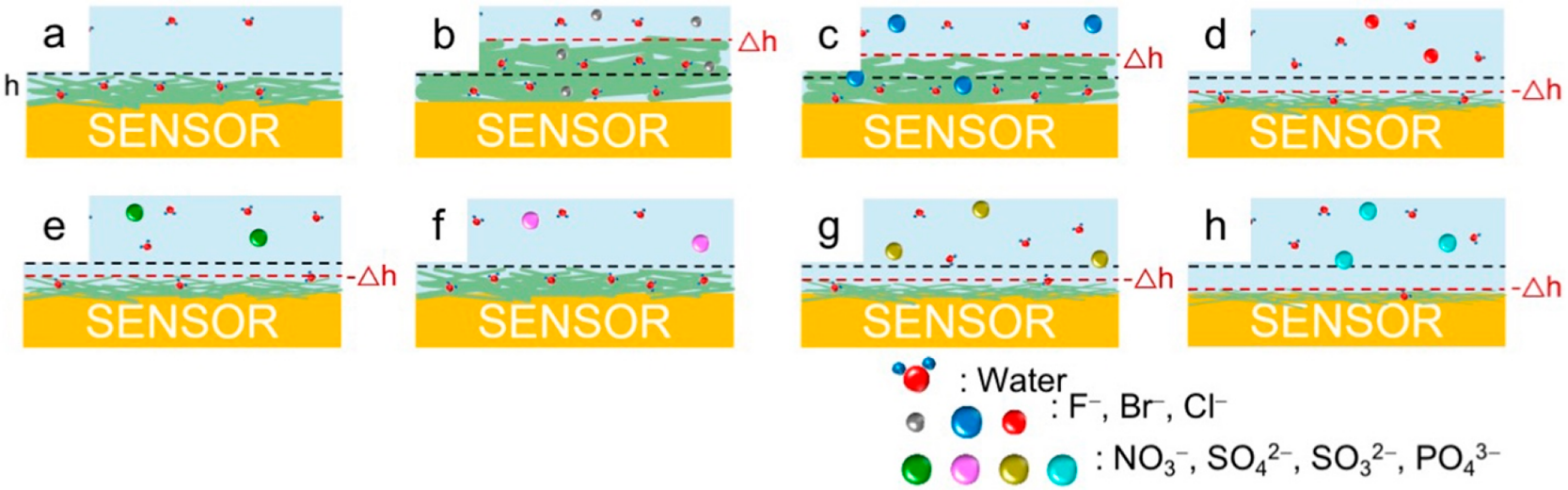
Schematic illustration displaying the effect of different anions on swelling/deswelling properties of NCh thin films. Each panel accounts for different conditions: (a) NCh is equilibrated in a salt-free environment; (b–d) NCh is exposed to halide anions (F^–^, Br^–^, and Cl^–^, respectively); (e–h) NCh is exposed to bulky and multivalent anions (NO_3_^–^, SO_4_^2–^, SO_3_^2–^, and PO_4_^3–^, respectively). The black and red dashed lines represent the thicknesses of the NCh coating before and after electrolyte exposure, respectively.

Salts with a higher ion valence are known^60^ to exhibit higher ionic strength upon dissociation in aqueous solutions compared to those with a lower ion valence. At a charged surface, the Debye length is strongly dependent on the ionic strength of the medium.^61^ Positively charged NCh is anticipated to interact and complex with available anions (either directly or indirectly depending on the type of anion) to screen the surface charge. Since one of the factors that dictates the overall efficiency and extent of charge screening is the ionic strength of the environment, the Debye length and the electric double layer (EDL) of NCh will be reduced to a greater extent in the presence of PO_4_^3–^, followed by SO_3_^2–^ and NO_3_^–^ at the same electrolyte concentration. Compression of the EDL translates into a reduction among NCh bionanoparticles, thereby permitting sufficient conformational change so that they can rearrange to lie in closer proximity. As this scenario occurs and becomes prominent, the NCh bionanoparticles can prefer to interact with themselves rather than with their surroundings (*i.e.*, water molecules). Moreover, due to unequal ion distribution and osmotic potential difference across the NCh film generated by the introduction of electrolytes, mobile (unbound) water molecules will prefer, for thermodynamic reasons, to leave NCh and, by so doing, mitigate such an imbalance. Consequently, deswelling caused by water removal is the most prevalent for trivalent anions relative to the other anions examined here at the same electrolyte concentration. The mechanism of anion-specific interaction and resulting NCh film swelling or deswelling behavior during QCM-D measurements is schematically represented in Figure 7.

## Conclusions

In this work, we have elucidated the effects of anion-specific interactions on NCh by exposing NCh thin films to various electrolytes and measuring the change in film thickness by QCM-D. According to dissipation energy measurements from QCM-D, the deposited NCh thin films remain stable and rigidly attached to the sensor in aqueous medium, even after repeatedly cycling the chamber with DI water and salt solutions of different concentrations. After accounting for salt-induced changes in the solution medium, corrected dissipation energy values confirm that the Sauerbrey condition is satisfied for all the tests performed here, in which case the similarly corrected change in frequency directly relates to changes in NCh film thickness. Different swelling behavior and NCh–anion interactions have been observed, especially when NCh is exposed to monovalent halide ions. The degree of swelling is sensitively dependent on the type of halide introduced. For example, the NCh film swells and remains swollen in the presence of F^–^ at all concentrations examined, whereas NCh initially swells at low concentrations, but eventually deswells at higher concentrations, of Br^–^. The thickness of NCh films monotonically decreases, and the films consistently deswell, when exposed to Cl^–^ anions. Similar behavior is observed when a bulky monovalent anion, NO_3_^–^, is substituted. The effect of monovalent anions on NCh film swelling follows a modified Hofmeister sequence in the order F^–^ > Br^–^ > NO_3_^–^ ≈ Cl^–^. Unlike the monovalent halides, however, bulky and multivalent anions systematically promote mass loss and thickness reduction with increasing electrolyte concentration. Anions possessing a higher valence state induce the largest mass loss over the entire concentration range examined, which can be explained in terms of the ionic strength of the electrolyte solutions.

We have also discovered that SO_4_^2–^ has negligible influence on NCh thin films as it causes little to no mass change at all concentrations. While this unexpected observation requires additional study, we speculate that it is related to the increased bulkiness and uniform charge distribution of SO_4_^2–^ compared to SO_3_^2–^. In summary, NCh interacts differently with anions varying in valence, electronegativity, and size, and such interactions dictate the swelling behavior of NCh films in the following order: F^–^ > Br^–^ > SO_4_^2–^ > NO_3_^–^ ≈ Cl^–^ > SO_3_^2–^ > PO_4_^3–^. We anticipate that this insight can further elucidate and extend the general applicability of the Hofmeister series in describing electrolyte interactions with charged bionanoparticles, as well as be used in a fashion analogous to cellulose nanocrystals^37^ to engineer structured films from electrolyte suspensions. Moreover, different (de)swelling behavior observed upon introduction of Na-coupled anions can be useful in various technologies that require precise regulation of water content. For example, deswelling can be beneficial in systems that require rapid coagulation to fabricate wet-spun filaments or 3-D printing paste. Furthermore, the presence of anions can alter not only the interactions between water and NCh but also the interactions between NCh and anions, which can promote substantial changes in material properties (*e.g.*, flow behavior, network formation, gel strength, and mechanical integrity). A fundamental understanding of these interactions will greatly broaden the utility of NCh in various application areas, such as, but not limited to, the biomedical, pharmaceutical, and polymer-composite fields.

## Supporting Information Available

Additional QCM data acquired from NCh films and uncoated (pristine) sensors in the presence of all the electrolytes examined here and used in the calculations provided in the main text (PDF)
