# Supplementary material for: Anion-Specific Water Interactions with Nanochitin: Donnan and Osmotic Pressure Effects as Revealed by Quartz Microgravimetry
Source: Langmuir. 2021 Sep 14;37(38):11242–50. doi: 10.1021/acs.langmuir.1c01585 (PMC8516332; doi:10.1021/acs.langmuir.1c01585)
Supplement: Supplementary file 1 — la1c01585_si_001.pdf [file la1c01585_si_001.pdf]

Supporting Information

Anion-Specific Water Interactions with Nanochitin: Donnan and Osmotic Pressure Effects as Revealed by Quartz Microgravimetry

Soo-Ah Jin,[1] **Saad A. Khan**,[1] **Richard J. Spontak**,[1,2] and **Orlando J. Rojas**,[1,3,4]

[1]Department of Chemical & Biomolecular Engineering, North Carolina State University, Raleigh, NC 27695, USA

[2]Department of Materials Science & Engineering, North Carolina State University, Raleigh, NC 27695, USA

[3]Bioproducts Institute, Departments of Chemical & Biological Engineering, Chemistry and Wood Science University of British Columbia, Vancouver V6T 1Z3, Canada

[4]Department of Bioproducts and Biosystems, Aalto University, Espoo 02150, Finland

In this **Supporting Information**, we provide additional QCM-D data that are introduced, analyzed and interpreted in the main text.

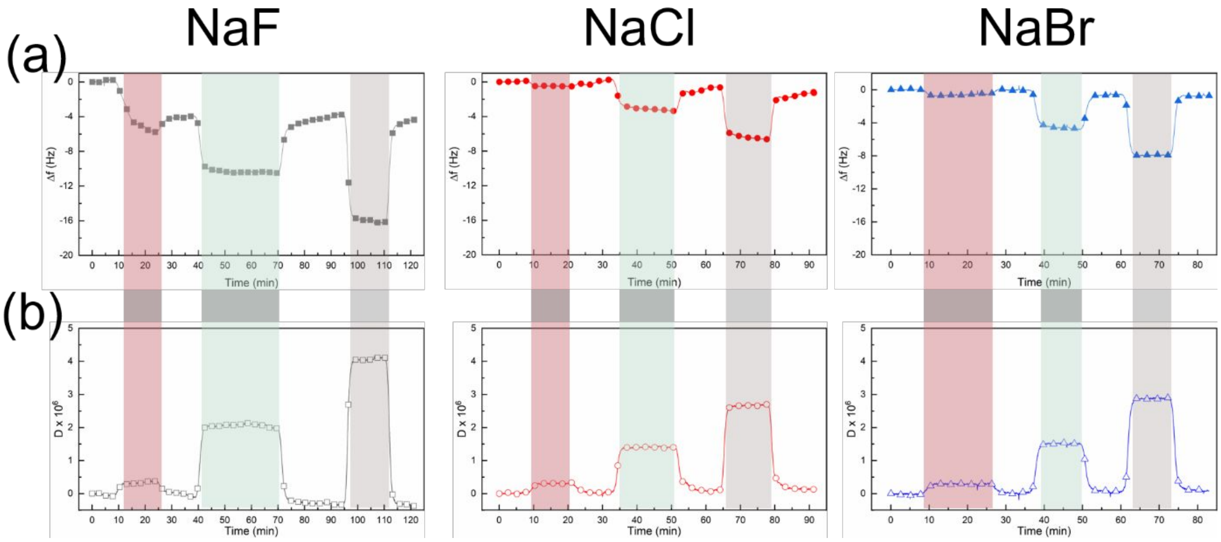

Figure S1. Representative QCM curve (3rd overtone) showing the change in (a) frequency and (b) energy dissipation for NCh thin films in the presence of NaF, NaCl and NaBr at different concentration. Red, green and grey shades indicate flow of 10 mM, 100 mM and 200 mM of salt solution, respectively. Milli-Q water is flowed through the chamber for rinsing purpose each time after salt addition.

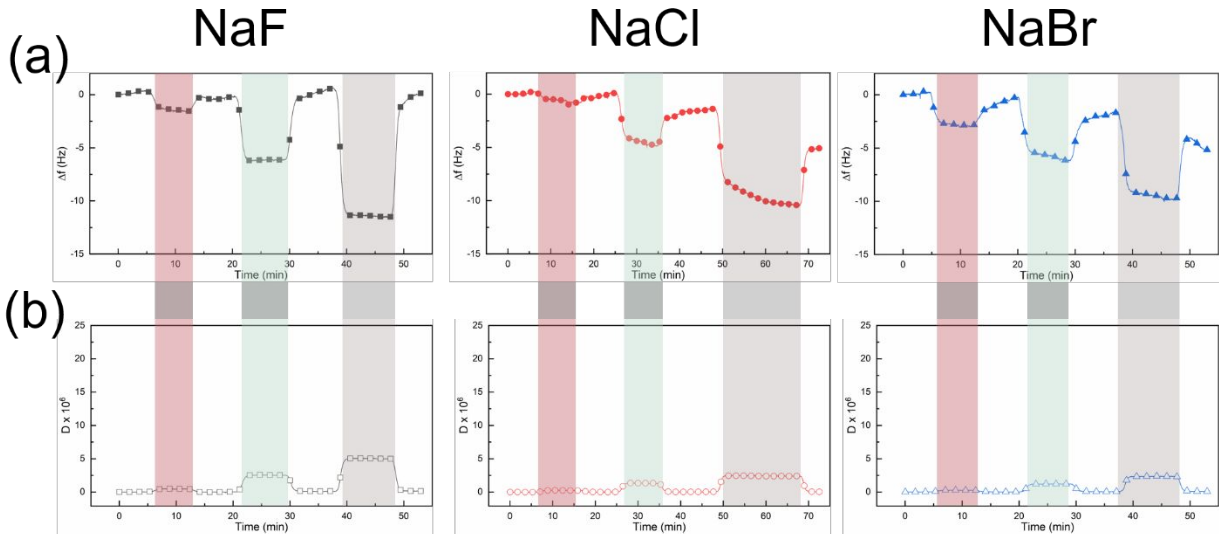

Figure S2. Representative QCM curve (3rd overtone) showing the change in (a) frequency and (b) energy dissipation for pristine sensors in the presence of NaF, NaCl and NaBr at different concentration to determine the bulk effect. Red, green and grey shades indicate flow of 10 mM, 100 mM and 200 mM of salt solution, respectively. Milli-Q water is flowed through the chamber for rinsing purpose each time after salt addition. The corresponding average value at each concentration was used to calculate the net change in frequency and dissipation energy.

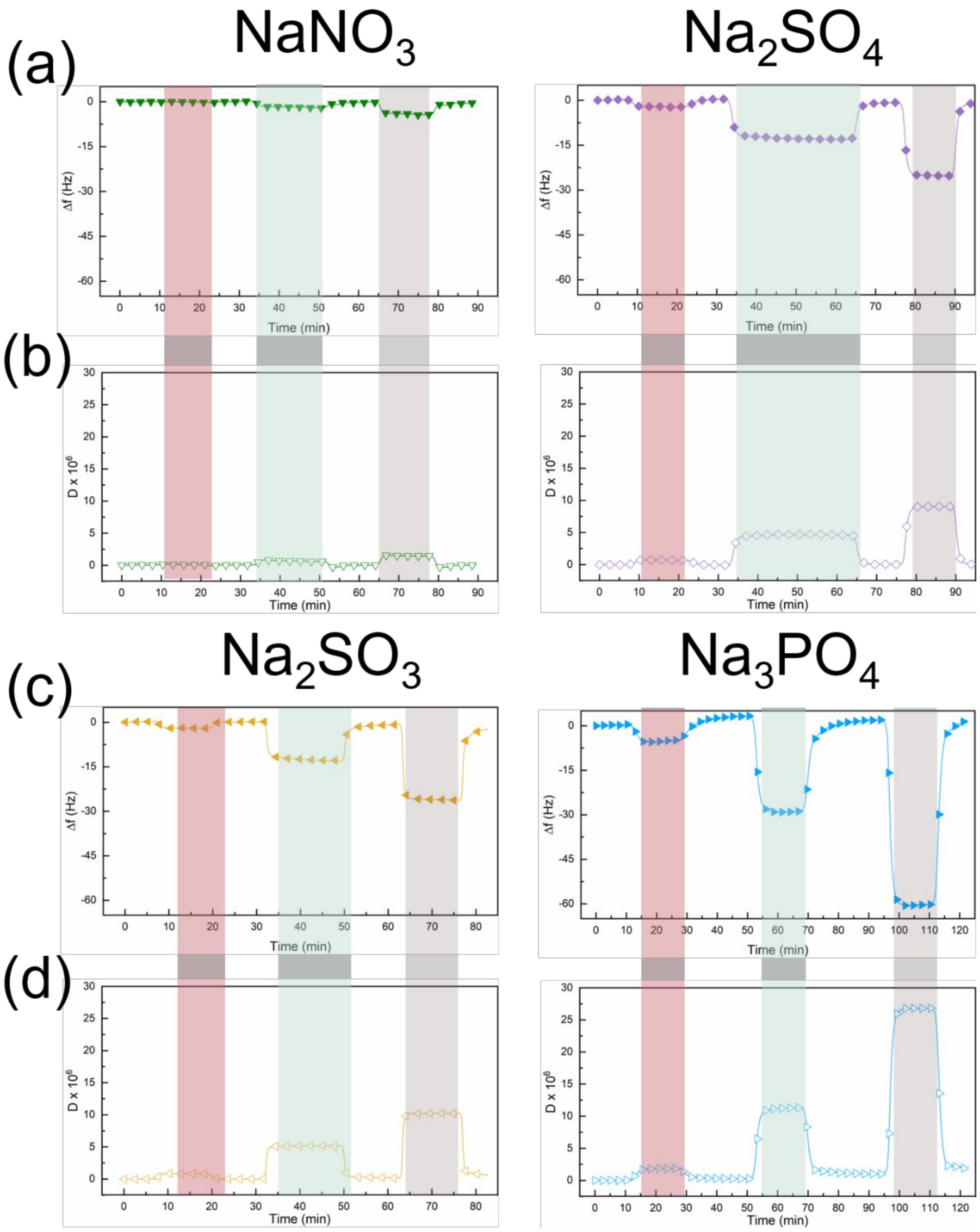

Figure S3. Representative QCM curve (3rd overtone) showing the change in (a, c) frequency and (b, d) energy dissipation for NCh thin films in the presence of $NaNO_3$, Na_2SO_4, Na_2SO_3 and Na_3PO_4 at different concentration. Red, green and grey shades indicate flow of 10 mM, 100 mM and 200 mM of salt solution, respectively. Milli-Q water is flowed through the chamber for rinsing purpose each time after salt addition.

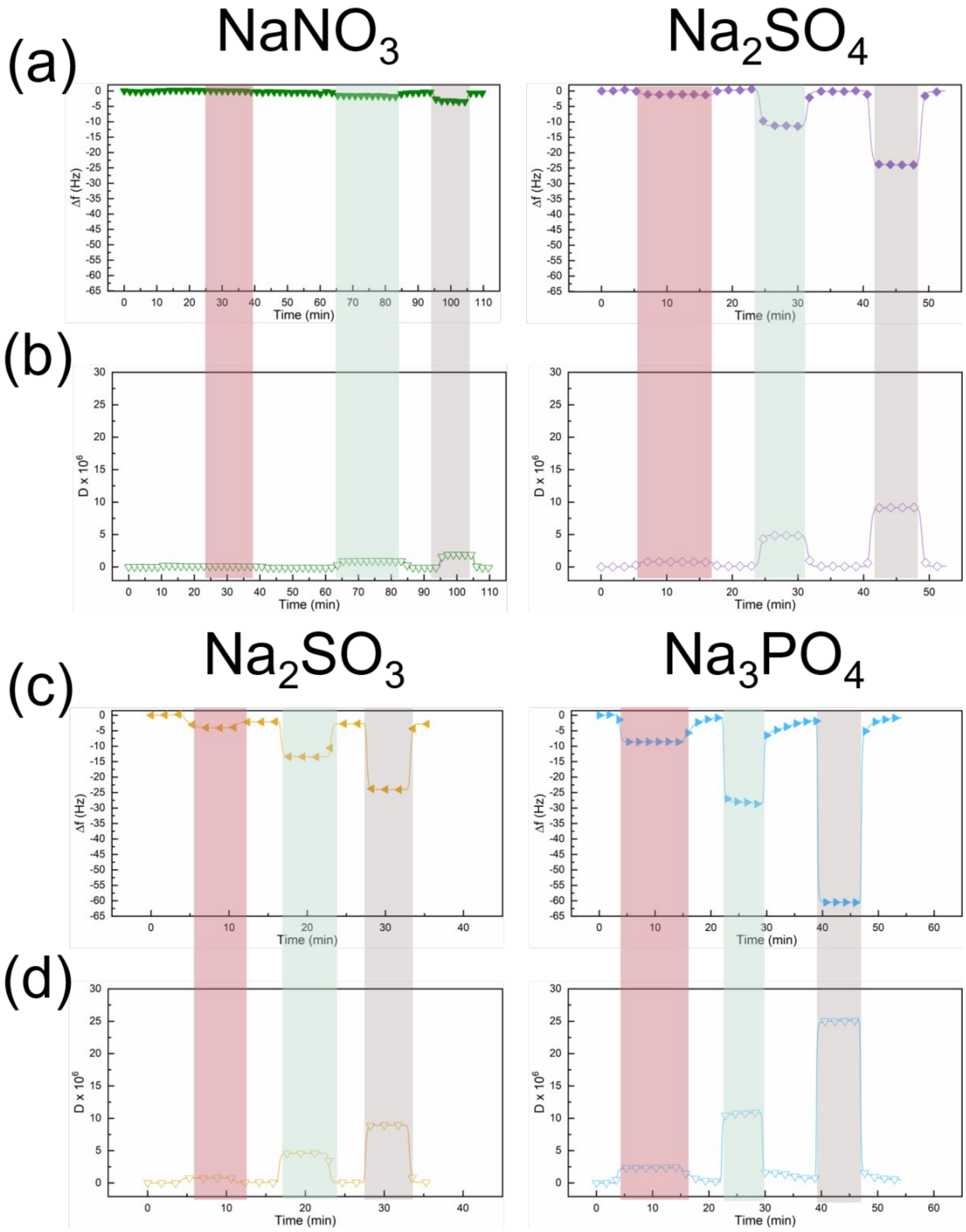

Figure S4. Representative QCM curve (3rd overtone) showing the change in (a, c) frequency and (b, d) energy dissipation for pristine sensors in the presence of $NaNO_3$, Na_2SO_4, Na_2SO_3 and Na_3PO_4 at different concentration to determine the bulk affect. Red, green and grey shades indicate flow of 10 mM, 100 mM and 200 mM of salt solution, respectively. Milli-Q water is flowed through the chamber for rinsing purpose each time after salt addition. The corresponding average value at each concentration was used to calculate the net change
